# Invasiveness of Ventilation Therapy Is Associated to Prevalence of Secondary Bacterial and Fungal Infections in Critically Ill COVID-19 Patients

**DOI:** 10.3390/jcm11175239

**Published:** 2022-09-05

**Authors:** Marie Louise de Hesselle, Stefan Borgmann, Siegbert Rieg, Jörg Janne Vehreshild, Christoph D. Spinner, Carolin E. M. Koll, Martin Hower, Melanie Stecher, Daniel Ebert, Frank Hanses, Julia Schumann, on behalf of the SAREL Investigators

**Affiliations:** 1 University Clinic and Outpatient Clinic for Anesthesiology and Operative Intensive Care, University Medicine Halle (Saale), 06112 Halle (Saale), Germany; 2 Department of Infectious Diseases and Infection Control, Ingolstadt Hospital, 85049 Ingolstadt, Germany; 3 Department of Medicine II, University of Freiburg, 79106 Freiburg, Germany; 4 Department II of Internal Medicine, Hematology and Oncology, Goethe University Frankfurt, 60323 Frankfurt, Germany; 5 Department I of Internal Medicine, Center for Integrated Oncology Aachen Bonn Cologne Duesseldorf, Faculty of Medicine and University Hospital Cologne, University of Cologne, 50931 Cologne, Germany; 6 German Center for Infection Research (DZIF), Partner Site Bonn-Cologne, 50937 Cologne, Germany; 7 Department of Internal Medicine II, University Hospital Rechts Der Isar, School of Medicine, Technical University of Munich, 81675 Munich, Germany; 8 German Center for Infection Research (DZIF), 38106 Brunswick, Germany; 9 Department of Pneumology, Infectious Diseases, Internal Medicine and Intensive Care, Klinikum Dortmund GmbH, 44137 Dortmund, Germany; 10 Emergency Department and Department for Infection Control and Infectious Diseases, University Hospital Regensburg, 93053 Regensburg, Germany

**Keywords:** COVID-19, SARS-CoV-2, multidrug-resistant pathogens, bacterial infections, fungal infections, secondary infections, intensive care medicine, ventilation, ECMO

## Abstract

Superinfections are a fundamental critical care problem, and their significance in severe COVID-19 cases needs to be determined. This study analyzed data from the Lean European Open Survey on SARS-CoV-2-Infected Patients (LEOSS) cohort focusing on intensive care patients. A retrospective analysis of patient data from 840 cases of COVID-19 with critical courses demonstrated that co-infections were frequently present and were primarily of nosocomial origin. Furthermore, our analysis showed that invasive therapy procedures accompanied an increased risk for healthcare-associated infections. Non-ventilated ICU patients were rarely affected by secondary infections. The risk of infection, however, increased even when non-invasive ventilation was used. A further, significant increase in infection rates was seen with the use of invasive ventilation and even more so with extracorporeal membrane oxygenation (ECMO) therapy. The marked differences among ICU techniques used for the treatment of COVID-19-induced respiratory failure in terms of secondary infection risk profile should be taken into account for the optimal management of critically ill COVID-19 patients, as well as for adequate antimicrobial therapy.

## 1. Introduction

COVID-19, a pulmonary disease from an infection with the single-stranded RNA virus SARS-CoV-2, has evolved into a global pandemic since March 2020. Clinical manifestation is highly variable. Asymptomatic courses, mild respiratory diseases, severe pneumonia, and severe organ dysfunction that can be accompanied by shock and death have been described [1,2,3,4,5,6]. A certain proportion of patients develop an increased respiratory rate (>30/min), a decrease in oxygen saturation with hypoxemia, and respiratory insufficiency, which requires intensive care, usually due to dyspnea [3,4,5,6,7].

During the course of the pandemic, specific therapies for COVID-19 have been developed. However, the corresponding drugs should be applied within the first days after infection [8]. Relative risk reduction with respect to hospitalization or adverse outcome by the administration of antivirals or neutralizing monoclonal antibodies was described with the initiation of therapy at 3 to a maximum of 6 days after symptom onset [8]. Therefore, in an intensive care unit (ICU), only supportive treatment options are available to alleviate symptoms. In severe respiratory failure, intubation and invasive ventilation is the standard therapy in clinical practice [9]. It is a life-saving measure and usually ensures a safe airway and sufficient oxygenation, along with carbon dioxide elimination [9]. Early intubation counteracts the progressive deterioration of lung function due to increased respiratory stress [4,6]. It has also been reported that the critical delay of intubation in the event of failure of non-invasive ventilation options is associated with a poorer prognosis [4,6]. However, invasive ventilation may be the cause of ventilator-associated lung injury [6,7,9]. In addition, the safe airway required for invasive ventilation can promote serious, even lethal, infections [10]. The scientific literature, therefore, also contains reports recommending the avoidance of intubation as long as it is not essential [11].

Patients with viral infections are known to be predisposed to secondary infections [12,13,14,15,16]. In particular, bacteria may benefit from viral infections, and even those that are normally harmless could turn into opportunistic pathogens. The viral facilitation of bacterial pathogenesis is based on complex and multifactorial processes that, ultimately, promote bacterial adherence, disrupt epithelial layers, lead to the displacement of commensal bacteria, and subvert the host immune response [16]. There are multiple reports associating SARS-CoV-2 with co-infections of, primarily, bacterial but also fungal origin. The most common bacterial microorganisms in respiratory cultures from COVID-19 patients are *Pseudomonas aeruginosa*, *Klebsiella* species, *Staphylococcus aureus*, *Escherichia coli*, and *Stenotrophomonas maltophilia* [13,14]. The main fungal pathogens identified are *Aspergillus* and *Candida* species, but there are also reports of secondary infections with *Mucormycetes*, *Histoplasma* spp, *Cryptococcus* spp, and *Pneumocystis jirovecii* [12]. Alarmingly, such secondary infections have been linked to a severe clinical course with possible poor outcome [15,16].

Infections are a common problem in ICUs. A critical condition, an impaired immune response, and invasive treatments (i.e., mechanical ventilation and catheterization) all pose risk factors for nosocomial infections [10,17,18,19,20,21]. It is of concern that secondary infections in viral diseases of the respiratory tract, such as influenza, have been described as causes of morbidity and mortality [22,23,24,25]. However, the prevalence and clinical impact of healthcare-associated infections of bacterial or fungal nature in COVID-19 patients treated in ICUs is not well-understood and constitutes a serious knowledge gap. There is also insufficient knowledge on whether bacterial colonialization present on admission impacts disease severity and outcome. More data on community-acquired colonializations, as well as nosocomial infections in ICUs, are needed to optimize the management and treatment of the most severe COVID-19 cases. This could not only help to save lives but also to improve antimicrobial stewardship [26,27,28,29].

The aim of the present study is to unravel the prevalence of community-acquired colonializations with multidrug-resistant bacteria, as well as healthcare-associated secondary bacterial and fungal infections, in critically ill COVID-19 patients treated at an ICU. The primary objective was to determine whether (i) there is an association between a patient’s infection status and the ventilation therapy used and whether (ii) co-infections are related to mortality. The secondary objectives are to examine the frequency of use and the clinical benefit of antimicrobial therapy in critically ill COVID-19 patients.

## 2. Materials and Methods

### 2.1. Patient Cohort

This study analyzed patient data from the Lean European Open Survey on SARS-CoV-2-Infected Patients (LEOSS) cohort [30]. The LEOSS project represents a non-interventional, multicenter network that aims at addressing the lack of in-depth knowledge on the epidemiology and clinical course of COVID-19. Established in March 2020, the LEOSS registry encloses data mainly on hospitalized COVID-19 patients. In the LEOSS protocol, patients can be included via PCR confirmed diagnosis or rapid antigen tests as an acceptable alternative. Detailed information on LEOSS can be found on the project’s website (https://leoss.net, accessed date: 5 August 2022). The study was registered at the German Clinical Trials Register (DRKS, No S00021145).

Clinical data are reported in an electronic case report form (eCRF) using the online platform ClinicalSurveys.net, which was developed by the University Hospital of Cologne (UHC), Germany, and is hosted by QuestBack, Oslo, Norway, on servers of the UHC [31]. Anonymized patient data are added to the LEOSS registry retrospectively at the end of the acute treatment setting, i.e., when either the treatment is completed or the patient has died. In order to ensure anonymity in all steps of the analysis process, an individual LEOSS Scientific Use File (SUF) was created, which is based on the LEOSS Public Use File (PUF) principles described in Jakob et al. [31]. Re-identification is prevented by vertical (categorical assessment of numerical variables) and horizontal data aggregation (data aggregation within the phases of disease). Categorization is based on four phases, which can be roughly characterized as asymptomatic or mild symptoms (uncomplicated phase), need for oxygen supplementation (complicated phase), need for critical care (critical phase), and the recovery phase. A detailed description of the clinical phases as defined in the LEOSS registry, as well as of the recorded data items, can be found on the project’s website (https://leoss.net; accessed on 4 August 2022) and in [32].

### 2.2. Study Design

This analysis included data of 840 patients who were documented by a LEOSS partner site between 23 March 2020 and 12 October 2020 due to COVID-19 disease diagnosed and treated between February 2020 and October 2020. Only patients who reached the critical phase according to the definitions of the LEOSS database [32] during the course of their COVID-19 disease were included in the analysis. The onset of the critical phase was declared if at least one of the following criteria was present: need for catecholamines, life-threatening cardiac arrhythmia, need for unplanned mechanical ventilation (invasive or non-invasive), prolongation (>24 h) of planned mechanical ventilation, liver failure with Quick <50% or INR >3.5, a qSOFA score of ≥2, or acute renal failure with need of dialysis. Dedicated intensive care data items were developed by a working group of specialized intensive care physicians (LEOSS Intensive Care Group) and implemented in the LEOSS registry. From this set, the following data items were analyzed: (i) the colonialization status of the patients with regard to multidrug-resistant pathogens at baseline, i.e., day of positive SARS-CoV-2 diagnosis (multidrug-resistant, Gram-negative bacteria (3MRGN/4MRGN), methicillin-resistant *Staphylococcus aureus* (MRSA), and vancomycin-resistant enterococci (VRE)), as well as bacterial and fungal superinfections in the critical phase; (ii) the ventilation treatments performed (non-invasive ventilation, invasive ventilation, or extracorporeal membrane oxygenation (ECMO)); (iii) the medications used; and (iv) the outcome (recovery or death). 3MRGN and 4MRGN are enterobacteriaceae, *Pseudomonas aeruginosa*, and *Acinetobacter baumannii* exhibiting resistance to three or four of these antibiotics or antibiotic groups: piperacillin, carbapenems, quinolones, and cephalosporins of the third generation. Two endpoints were defined: (i) the prevalence of community-acquired colonializations and healthcare-associated secondary infections in patients in need of or receiving a specific ventilation therapy (non-invasive ventilation, invasive ventilation, or ECMO) and (ii) the effect of community-acquired colonializations and healthcare-associated secondary infections on patient outcome.

### 2.3. Statistical Analysis

All data were presented as categorical variables (numbers and percentages). To compare categorical variables, Pearson’s chi-squared or Fisher’s exact test was used where appropriate. The level of significance was set at *p* < 0.05. The data management, statistical analysis, and computation of figures were conducted using R (R Development Core Team, Vienna, Austria, Version 4.1.1, 2021).

## 3. Results

### 3.1. Characteristics of the Study Population

From February 2020 to October 2020, 840 SARS-CoV-2-positive diagnosed patients were admitted to an ICU at a LEOSS study site (Table 1). The majority of the patients were between 46 and 85 years old (85.1%; 715/840), and 6.7% (56/840) were older than 85 years. A total of 602 of the 840 patients (71.7%) were male; the only age group without a male predominance was the 85+ age group. The most common comorbidities were hypertension (61.0%, 512/840), diabetes mellitus (28.1%; 236/840), chronic kidney disease (17.3%; 145/840), coronary artery disease (16.7%; 140/840), and atrial fibrillation (16.0%; 134/840). Only 13.9% of the patients (117/840) had no documented comorbidities; one comorbidity was documented for 22.0% of the patients (185/840), and multiple comorbidities (up to 14) were reported in 64.1% of the patients (538/840). Mechanical ventilation therapy was used in the vast majority of patients. In 21.5% of the patients (181/840), an attempt at non-invasive ventilation failed, requiring intubation; in 37.0% of the patients (311/840), intubation was performed without prior non-invasive ventilation. Exclusive non-invasive ventilation was documented in 10.4% of the patients (87/840). Extracorporeal membrane oxygenation (ECMO) was required in 13.6% of the patients (114/840). Still, 17.5% of the patients (147/840) did not receive mechanical ventilation therapy. The majority of the patients (66.2%, 556/840) had a length of stay in the ICU of 0–3 weeks; 264 of 840 patients (31.4%) received intensive care for 4–9 weeks, and for 2.4% of the patients (20/840), a length of treatment in the ICU exceeding 9 weeks was documented. The overall mortality rate was 46% (386/840).

### 3.2. Community-Acquired Colonializations with Multidrug-Resistant Bacteria

Complete or at least partial information on colonializations with multidrug-resistant pathogens at baseline (say of positive SARS-CoV-2 diagnosis) was available for 71.2% of the patients (598/840). Among these, colonialization with 3MRGN was documented in 2.8% of the cases, with MRSA in 2.6% of the cases and VRE in 4.1% of the cases. However, the majority of the patients (75.1% of the cases) were declared free of colonialization with these bacteria on presentation. Information on 4MRGN was captured in the dataset, but so few infections were reported that details were not made available in the LEOSS Scientific Use File to maintain patient anonymity.

**Figure 1:**
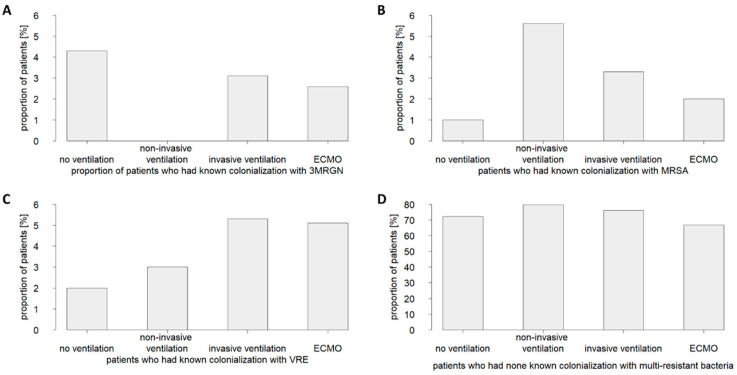
Prevalence of community-acquired colonializations with multidrug-resistant bacteria in patients critically ill with COVID-19 who received no ventilation therapy or were treated with non-invasive ventilation, invasive ventilation, or ECMO (extracorporeal membrane oxygenation). Shown are the proportions of patients who were colonized with (**A**) 3MRGN (multidrug-resistant Gram-negative bacteria), (**B**) MRSA (methicillin-resistant *Staphylococcus aureus*), or (**C**) VRE (vancomycin-resistant enterococci), or those where (**D**) no colonization was found.

Examining in detail the patient subcohorts grouped by ventilation therapy performed (i.e., no ventilation, non-invasive ventilation, invasive ventilation, or ECMO) indicated no fundamental differences in colonialization prevalence with multidrug-resistant pathogens (Figure 1). Thus, the data did not support the hypothesis that a community-acquired colonialization with a multidrug-resistant pathogen increased the risk of a critically ill COVID-19 patient to require invasive ventilation or ECMO therapy.

**Figure 2:**
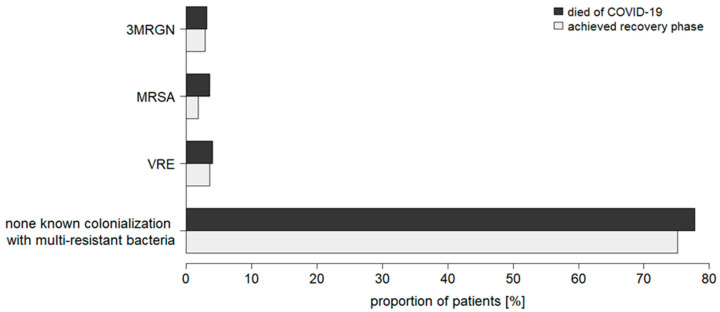
Prevalence of community-acquired colonializations with multidrug-resistant bacteria in recovered and deceased patients critically ill with COVID-19 (total cohort). MRGN: multidrug-resistant Gram-negative bacteria; MRSA: methicillin-resistant *Staphylococcus aureus*; VRE: vancomycin-resistant enterococci.

Furthermore, the data demonstrated no association between a pre-existing colonialization with a multidrug-resistant bacterium and mortality in critically ill COVID-19 patients (Figure 2). No significant difference in colonialization status was observed between recovered and deceased patients (Figure 2).

### 3.3. Healthcare-Associated Bacterial and Fungal Infections

Information on hospital-acquired bacterial and fungal infections of critically ill patients in the ICU was available for 806 cases (96.0% of the total cohort). Overall, secondary bacterial infection was documented for 326 patients in the critical phase (40.4% of the cases), and secondary fungal infection was documented for 118 patients in the critical phase (14.6% of the cases).

**Figure 3:**
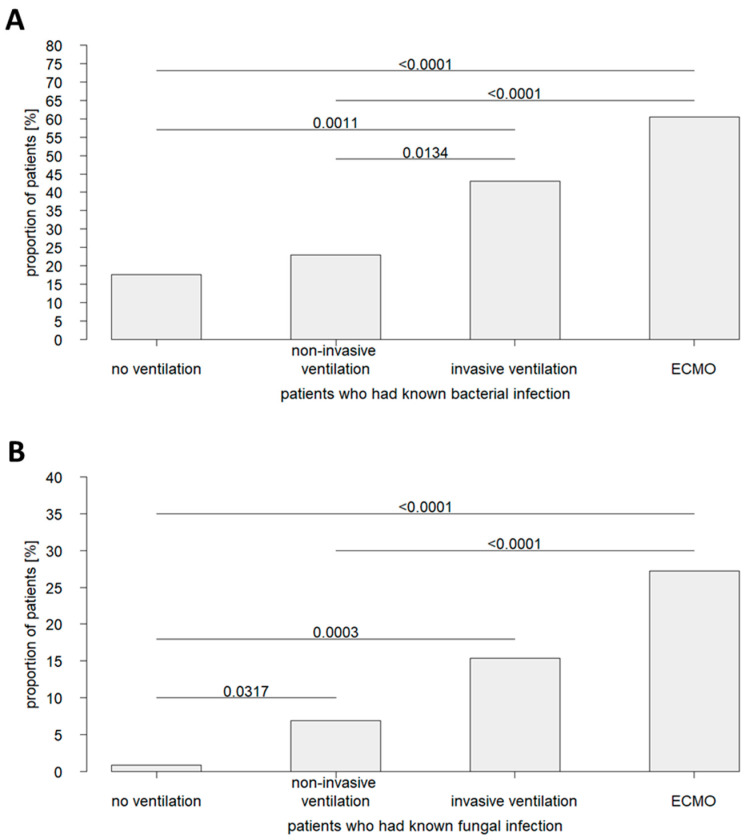
Prevalence of secondary infections in patients critically ill with COVID-19 who received no ventilation therapy or were treated with non-invasive ventilation, invasive ventilation, or ECMO. Shown are the proportions of patients with (**A**) bacterial and (**B**) fungal infections of nosocomial origin.

Remarkably, a comparative analysis of patient cohorts subdivided by ventilation therapy revealed significant differences in infection status (Figure 3). Healthcare-associated secondary infections with bacteria or fungi had an above-average prevalence in ECMO patients (bacterial co-infections in 60.5% of cases and fungal co-infections in 27.5% of cases). As such, ECMO patients were affected by nosocomial infections more frequently than invasively ventilated patients, in whom secondary co-infections with bacteria were documented in 43.1% of cases and with fungi in 15.4% of cases. However, a further lower, below-average prevalence of nosocomial infections was reported for the cohort of non-invasively ventilated patients (secondary bacterial co-infections in 23.0% of cases and secondary fungal co-infections in 6.9% of cases). In patients who did not receive ventilation, hospital-acquired bacterial co-infections were seen in 17.7% of cases and fungal co-infections in 0.9% of cases. These data support the hypothesis that invasive therapy procedures accompany an increased risk for healthcare-associated infections.

**Figure 4:**
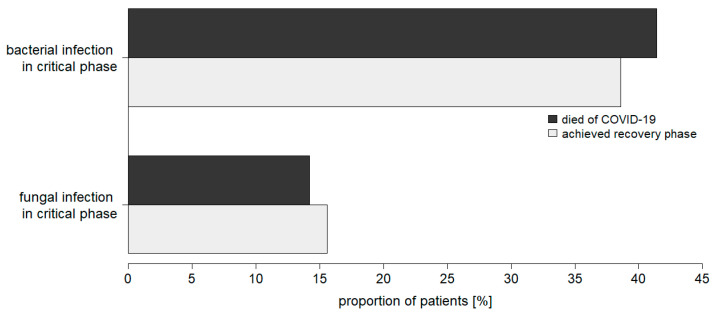
Prevalence of secondary bacterial and fungal infections in recovered and deceased patients critically ill with COVID-19 (total cohort).

There were no significant differences in the frequencies of secondary bacterial or fungal infections when comparing critically ill COVID-19 patients who died or reached the recovery phase (Figure 4). Thus, no effect of hospital-acquired infections on outcome became apparent.

### 3.4. Antimicrobial Therapy: Frequency of Use and Clinical Benefit

**Figure 5:**
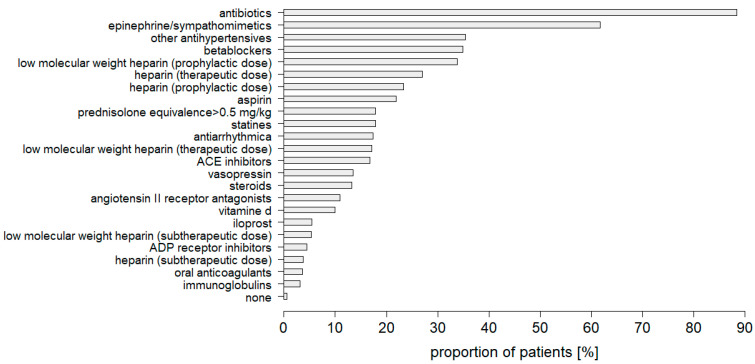
Medication used in intensive care for patients critically ill with COVID-19.

Antibiotic use data were available for only 285 critically ill patients with COVID-19 treated in the ICU (33.9% of the total cohort), an alarmingly low figure in terms of antimicrobial stewardship. An in-depth review of the pharmacologic treatment of these patients found that antibiotic treatment was the most frequently administered medication, even preceding epinephrine and sympathomimetics (Figure 5). Therefore, antibiotic therapy was administered in the vast majority of cases (88.4%), although bacterial infection was documented in just 40.4% of the overall patients and 47.6% of this particular patient subset.

A detailed examination of the patient cohorts subdivided by ventilation therapy revealed that almost all the ECMO patients (95.7% of cases) received antibiotic treatment. In the case of invasive ventilation, antibiotics were administered in 91.8% of the patients. Substantially less frequently, but still at a high level, antibiotics were used in non-invasively ventilated patients (71.9% of cases) and patients who did not receive ventilation therapy (75.0% of cases).

There was no difference in antibiotic use frequency between patients who died as a result of COVID-19 infection and those who reached the recovery phase. However, considering the high rate of antibiotic use, especially in intubated patients and patients on ECMO therapy, no valid conclusion can be drawn from this as to the clinical benefit of antibiotic treatment. While empiric antibiotic treatment might prevent the development of nosocomial infections, it also impedes microbial pathogen detection and, therefore, hinders specific anti-infective therapy when needed.

## 4. Discussion

In the present study, the relations between the colonization with nosocomial bacteria, the rate of nosocomial infections, the necessity to undergo ventilation, and the mode of ventilation were examined for COVID-19 patients treated in an ICU. The results of the study showed that colonializations with 3MRGN, MRSA, and VRE were similar in non-ventilated patients and patients undergoing non-invasive, invasive, and oxygenation ventilation, indicating that colonization was not associated with ventilation or its invasiveness. Moreover, a colonization with multi-resistant bacteria was not associated with a fatal outcome. On the other hand, the number of nosocomial infections significantly correlated with the invasiveness of the ventilation modus, indicated by the finding that the lowest infection rates were observed in non-ventilated COVID-19 patients, while the highest numbers occurred in patients oxygenated with ECMO. However, these infections were not related with a fatal outcome.

Since the outbreak of the pandemic, the field of COVID-19 has evolved. Vaccines have been developed that are proven to reduce the need for ICU treatment in the case of a breakthrough infection [33]. Furthermore, there are now drug treatment strategies that, when initiated in a timely manner, can have a mitigating effect on disease severity and, thus, counteract the need for critical care [8]. However, a significant number of patients still develop respiratory insufficiency requiring admission to an ICU and targeted ventilation. The present study clearly demonstrated that such treatment was associated with an increased risk of secondary infections, with the invasiveness of the ventilation technique used being an influential variable. This is even more important as no correlation between patient characteristics, such as age or comorbidities, and the occurrence of secondary infections was found.

Infections pose a significant problem in ICUs [10,19,34,35,36], especially in patients with viral respiratory infections. In severe influenza, for example, bacterial co-infections have been described in up to 20% to 30% of cases, and superinfections have been associated with pronounced disease severity and a higher risk of death [17,26,28,29,34,35,36]. Consequently, in critically ill COVID-19 patients, the prevalence of bacterial and fungal co-infections, their impact on the clinical course, and appropriate antimicrobial therapy in a primarily viral disease are of particular importance.

Since the very beginning of the pandemic, co-infections of COVID-19 patients have been reported [18,19,35,36,37,38,39]. It needs to be noted that the several studies reporting superinfections have not distinctly distinguished between community-acquired and healthcare-associated infections, thus limiting the validity of these studies. Our study, however, clearly showed that the vast majority of patients had no evidence of colonialization with bacterial multidrug-resistant microorganisms at baseline, and only a single-digit percentage of patients was affected by colonialization with 3MRGN, MRSA, or VRE at hospital presentation. Thus, for the group of COVID-19 patients with a critical course, it resulted that colonization at the time of diagnosis of SARS-CoV-2 infection was rare, especially with regard to the most clinically relevant multidrug-resistant pathogens. Indeed, other studies have also reported low rates of early infection and, rather, direct the focus to nosocomial infection [17,34]. Reported rates of secondary bacterial infections in critically ill ICU patients with COVID-19 have ranged from 8.1% to 42.8% [13,17,18,34,35,36,37,38,39]. There is also a wide range of reported infection rates with respect to secondary fungal infections. As such, a meta-analysis of eight studies related to COVID-19 patients treated in an ICU setting reported an infection rate of 9.6% (95% CI 6.8–12.4) [37]. Specifically, in mechanically ventilated COVID-19 patients, a multicenter prospective cohort study found a rate of invasive fungal infections of 26.7% [40]. One can only speculate as to the causes of the wide range of case numbers reported. Workload, unfavorable physician- or nurse-to-patient ratios, and a lack of laboratory capacity, especially in the early months of the pandemic, might have partially limited the capability for widespread infection control. For additional consideration, especially for critically ill ICU patients, the true prevalence of secondary infections may be underestimated due to the untimely deaths of these patients. In any case, our study provided clear evidence that nosocomial infections of bacterial and fungal origin were common in COVID-19 patients receiving intensive care and warrant awareness and adequate management. There is a need for the proper diagnosis and effective treatment of not only bacterial but also fungal infections in COVID-19 patients receiving intensive care.

Critically ill COVID-19 patients undergo a variety of invasive interventions in the ICU, such as mechanical ventilation and catheterization, which promote bacterial and fungal infections [10,13,17,19,21,26,38,39,41] and are described to be more frequently subject to additive bacterial and fungal infections compared to patients treated in regular wards [13,35,36,37,38,39]. We were, therefore, interested in the impact of the level of therapeutic invasiveness on the prevalence of healthcare-associated infections. Indeed, our data clearly proved that non-ventilated ICU patients were at low risk for secondary infections. The risk of infection increased markedly, even when non-invasive ventilation was used. A dramatic rise in the proportion of patients with nosocomial infections was seen with the use of invasive ventilation, and even more so with ECMO therapy. Actually, in ECMO-treated patients, healthcare-associated bacterial infections were present in about two-thirds of cases and healthcare-associated fungal infections in nearly one-third of cases. Our data provided evidence that the techniques used in intensive care for the treatment of COVID-19-induced respiratory insufficiency differed significantly with respect to risk profiles for secondary infections. Based on these data, close infection control is recommended, especially when invasive methods are required.

There is ongoing discussion as to whether secondary infections impact mortality in COVID-19 patients. Some studies have reported an association of nosocomial infections with adverse outcome, whereas other studies have found no such correlation [19,20,37,39,41,42]. In our study, the rates of secondary infections of surviving and deceased COVID-19 patients were not significantly different. The same was true for community-acquired colonialization with 3MRGN, MRSA, and VRE. Patient-associated factors, such as pre-existing conditions, may be critical in determining whether co-infections ultimately impact survival. In a risk analysis, Silva et al. already showed that co-infections increased the risk of death, specifically in patients with obesity, cardiovascular disease, or diabetes mellitus [41]. Obesity, cardiovascular disease, and diabetes mellitus are known risk factors for a critical course of SARS-CoV-2 infection and are common in ICU patients (as in the present study cohort). This raises the possibility of a vicious circle. Large cohort studies are needed to investigate this in detail, with particular priority on ICU patients, given their high risk of developing secondary infections.

Several guidelines, such as those from the World Health Organization (WHO) and the Surviving Sepsis Campaign, advocate the use of empiric antibiotics in patients with severe COVID-19 [7,43]. This explains why, in our study, the absolute majority of patients (88.4%) were treated with antibiotics, despite the fact that only half of these patients had a positive finding of bacterial infection. Other studies have consistently reported hospitalized COVID-19 patients receiving antimicrobial therapy in 50% to 100% of cases [13,15,18,20,26,27,34,36,38]. The undifferentiated use of antimicrobial agents is known to increase selection pressure and may promote the spread of resistant bacterial strains. Indeed, there are concerns that the increased usage of antibiotics in the context of the COVID-19 pandemic may worsen the issue of multidrug-resistant pathogens worldwide [27,28,29,34]. Strict adherence to antibiotic stewardship programs, effective implementation of infection control procedures, and maintenance of established hygiene standards need to be upheld even in pandemic settings. This is particularly true for ICUs, as invasive treatments are key to the development of secondary infections, as illustrated by the present study.

Our study has certain limitations. Due to its retrospective nature, data availability was limited to the medical records added to the LEOSS registry. We did not have information of interest, such as the presence of antibiotic resistance or the type, dosage, and timing of antibiotic, antifungal, or immunosuppressant drugs. Accordingly, we could not make statements on these potential influencing factors. This study included ICU patients suffering from COVID-19 from Europe, predominantly Germany, which may limit the generalizability of our findings.

## 5. Conclusions

Healthcare-associated infections are common in critically ill COVID-19 patients treated in ICUs. Our study highlighted the importance of the type of intensive care treatment when it came to nosocomial infections. Patients receiving invasive ventilation had markedly increased rates of secondary bacterial and fungal infections compared with those receiving non-invasive treatment. Another distinct increase in infection rates was documented in ECMO-treated patients. This knowledge should inform future treatment decisions in the ICU.

## Figures and Tables

**Table 1:** Epidemiological data of the total cohort, as well as subcohorts, subdivided according to the type of ventilation performed. ECMO: extracorporeal membrane oxygenation.

	Total Cohort	Subcohort: No Ventilation	Subcohort: Non-Invasive Ventilation	Subcohort: Invasive Ventilation	Subcohort: ECMO
Patient count	840	147	87	492	114
Age range (years)	<1 to >85	<1 to >85	36 to >85	9 to >85	26 to 85
Gender distribution (male/female)	602/238	92/55	60/27	357/135	93/21
Number of comorbidities	0 to 14	0 to 14	0 to 11	0 to 12	0 to 7
Length of stay in ICU (weeks)	0 to 10	0 to 10	0 to 6	0 to 10	0 to 10
Length of ventilation (weeks)	up to 9	-	up to 6	up to 9	up to 9
Mortality rate (%)	46.0	53.7	39.1	41.1	62.3

## Data Availability

Patient data from the LEOSS registry are subject to the LEOSS governance, data use, and access policy (policy text available on https://leoss.net; accessed on 4 August 2022).
